# Development and Evolution of Unisexual Flowers: A Review

**DOI:** 10.3390/plants11020155

**Published:** 2022-01-07

**Authors:** Florian Jabbour, Felipe Espinosa, Quentin Dejonghe, Timothée Le Péchon

**Affiliations:** 1 Institut de Systématique Évolution Biodiversité (ISYEB), Muséum National d’Histoire Naturelle, CNRS, Sorbonne Université, EPHE, Université des Antilles, 57 rue Cuvier, CP39, 75005 Paris, France; dejonghe.quentin@outlook.fr; 2 Independent Researcher, Carrera 13 # 113-24, Bogotá 110111, Colombia; felipeespinosam@gmail.com; 3 Meise Botanic Garden, Nieuwelaan 38, 1860 Meise, Belgium; timothee.lepechon@plantentuinmeise.be; 4 Fédération Wallonie-Bruxelles, Service Général de l’Enseignement Supérieur et de la Recherche Scientifique, Rue A. Lavalée, 1, 1080 Brussels, Belgium

**Keywords:** cryptic dioecy, floral gender, functional sex, meristem, ontogenic pathway, sexual system

## Abstract

The development of unisexual flowers has been described in a large number of taxa, sampling the diversity of floral phenotypes and sexual systems observed in extant angiosperms, in studies focusing on floral ontogeny, on the evo-devo of unisexuality, or on the genetic and chromosomal bases of unisexuality. We review here such developmental studies, aiming at characterizing the diversity of ontogenic pathways leading to functionally unisexual flowers. In addition, we present for the first time and in a two-dimensional morphospace a quantitative description of the developmental rate of the sexual organs in functionally unisexual flowers, in a non-exhaustive sampling of angiosperms with contrasted floral morphologies. Eventually, recommendations are provided to help plant evo-devo researchers and botanists addressing macroevolutionary and ecological issues to more precisely select the taxa, the biological material, or the developmental stages to be investigated.

## 1. Introduction

The flower is the structure of angiosperms where female and male gametophytes are produced through meiosis. A flower including organs of both sexes is called bisexual, hermaphroditic (Box 1), or perfect. Except for a couple of genera (*Lacandonia* E. Martínez & Ramos (Triuridaceae), and *Trithuria* Hook. f. (Hydatellaceae), [1]), the set of stamens (i.e., the androecium) surrounds the gynoecium. The gynoecium consists of one or more unicarpellate pistils, or of one multicarpellate pistil.

Box 1 **Etymology of the term “hermaphrodite”.**

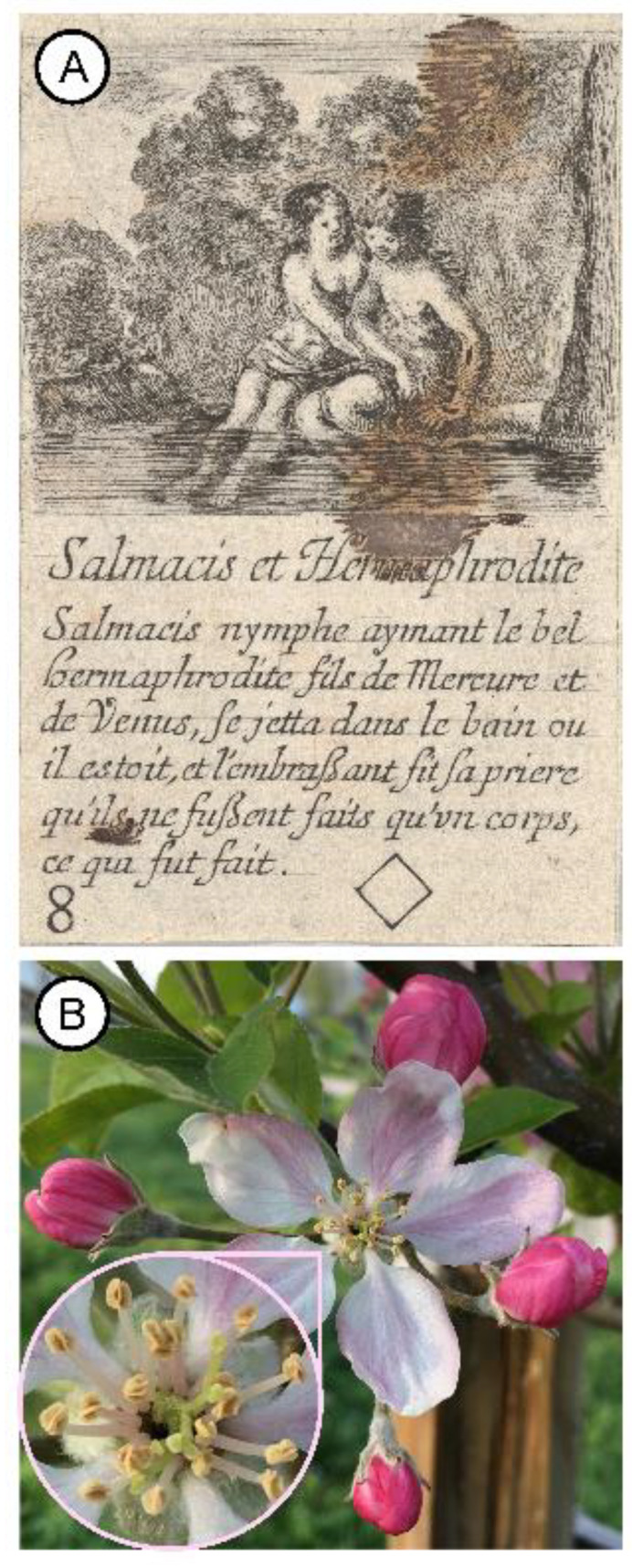

  The adjective “hermaphrodite” is built upon the names of the Greek gods Hermes and Aphrodite, who, according to the Metamorphoses by the Roman poet Ovid [2], gave birth to a child, named Hermaphrodite after both his parents. The handsome young man was swimming in a lake when the naiad Salmacis fell in love with him, although her feelings were not reciprocal. The gods heard Salmacis’ prayers to be forever united to her beloved one, and merged both people into a single body, exhibiting both male and female sexes and physical attributes (Figure A).  Hermaphrodite in mythology and in botany.  (A) Salmacis and Hermaphrodite, from the Game of Mythology, by Stefano della Bella, 1644 (Metropolitan Museum of Art, New York, https://www.metmuseum.org/art/collection/search/412360 (accessed on 18 December 2021), Bequest of Phyllis Massar, 2011). An English translation of the short text would be “Salmacis and Hermaphrodite. The nymph Salmacis loved the handsome Hermaphrodite, son of Mercury and Venus. She pushed him into the water, and while kissing him, her wish to be united with him in the same body was granted”.  (B) Hermaphroditic flower of *Malus domestica* (Suckow) Borkh. (Rosaceae; photograph: F. Jabbour). The numerous stamens surround the pentamerous gynoecium.

The majority of angiosperms bear bisexual flowers, and few flowering plants (~10% of the total number of species) have unisexual (Box 2) flowers [3].

Box 2 **Glossary.**

**Androdioecy/Gynodioecy**: Two contrasting sexual systems; a gynodioecious population consists of a mixture of female and hermaphroditic individuals. Gynodioecy is not uncommon. An androdioecious population is composed of male and hermaphroditic individuals. Androdioecy is very rare [4,5].

**Andromonoecy**/**Gynomonoecy**: An andromonoecious species is characterized by the fact that a given individual presents both male and bisexual flowers but no female flowers. A gynomonoecious species presents both female and bisexual flowers on the same plant, but no male flowers [6].

**Automimicry**: Imitation of male flowers by female flowers to attract pollinating insects searching for rewarding male flowers [7].

**Cosexual:** The most common sexual system in flowering plants, in which a population comprises a single sexual class of hermaphrodites (cosexes) and on average individuals reproduce equally through female and male function [4].

**Cryptic dioecy**: Flowers appear morphologically bisexual; however, only one of the two sexes is functional [8].

**Dichogamy**: Temporal difference in sexual organs maturation, thus promoting cross-pollination rather than self-pollination. Differences in the timing of pollen dispersal from anthers and stigma receptivity of flowers. In protandry, pollen is dispersed before stigmas are receptive, and in protogyny, stigmas are receptive before pollen is dispersed from anthers [3].

**Dioecy**: A sexual polymorphism in which populations contain female and male plants [3]. Sexual system of taxa in which archegonia/embryo sacs and antheridia/microsporangia are produced on each sporophyte [9,10].

**Dioicy**: The same, but for gametophytes. Archegonia and antheridia are produced on separate gametophytes [9,10].

**Gender**: Functional (rather than morphological) sex.

**Gender strategies:** Concern the femaleness and maleness of individuals and reflect the relative contributions to fitness from maternal and paternal investment [11].

**Herkogamy**: Male and female sexual structures (i.e., stigmas and anthers) are separated from each other in a flower. This configuration reduces the likelihood of self-pollination [3].

**Heterodichogamy**: Population-level dimorphism involving two morphs synchronously and reciprocally dichogamous, with male- or female-first phases [12].

**Inbreeding depression:** The reduction in viability and/or fertility of inbred offspring compared to outcrossed offspring as a result of the expression of deleterious recessive alleles in homozygous genotypes. Inbreeding depression is a key factor in determining mating system evolution. It is usually most strongly expressed when inbreeding occurs in a predominantly outcrossing species [4].

**Leaky dioecy**: The occasional occurrence of bisexual flowers in a few individuals of a dioecious population.

**Mating system:** The mode of transmission of genes from one generation to the next through sexual reproduction. Important determinants of plant mating systems are the ovule selfing rate and male fertility [11].

**Monoecy**: Sexual system of taxa in which archegonia/embryo sacs and antheridia/microsporangia are produced on each sporophyte [9,10].

**Monoicy**: The same, but for gametophytes. Archegonia and antheridia are produced on each gametophyte [9,10].

**Pistillate (male-sterile)**/**Staminate (female-sterile):** Flower with only female/male organs, respectively [6].

**Pistillode (carpellode):** A rudimentary sterile pistil [6].

**Polygamy**: Various combinations of sexual expression, such as andromonoecy or androdioecy [13]. Polygamy sensu Linnaeus [14] refers to the presence of unisexual and bisexual flowers on some or all individuals [10].

**Sexual Dimorphism:** Differences between the sexes in primary and secondary sex characters. The former related directly to male (androecium) and female (gynoecium) sexual organs, and the latter to differences between the sexes in structures other than sex organs themselves, including any aspect of morphology or physiology [15].

**Sexual polymorphism**: The co-occurrence within a single interbreeding population of morphologically distinct mating groups that are distinguished by differences in their sexual organs [3].

**Sexual system**: Gender expression and its occurrence at different levels (intrafloral, individual, population, or species levels) [12] (see Table 1).

**Staminode:** A sterile or abortive stamen, usually smaller than a stamen and not bearing mature pollen [6].

**Unisexual:** (Flowers) that have only functional male parts or female parts [6].

**Dicliny:** Spatial separation of sex in different flowers (sensu [16]), the flowers are therefore unisexual.

**Unisexual by abortion (flower of type I):** Initiation of androecial and gynoecial organs occurs in all flowers followed by the termination of development in one or the other organ set [17].

**Unisexual from inception (flower of type II):** The floral meristem initiates only androecial or gynoecial organs and does not go through a hermaphroditic stage [17].

This latter condition, known as dicliny (Box 2), is associated with a wide spectrum of gender (Box 2) systems that involve various combinations of female, male, and hermaphroditic flowers at the plant and population levels [3,18]. In his *Regnum Vegetabile Secundum Systema Sexuale* of his *Systema Naturae*, Linnaeus [14] (p. 23) distinguished hermaphroditic plants from plants having either male or female flowers. He used variation in sexual structures as the basis for plant classification. Linnaeus’ Monoecia (from Ancient Greek μόνος (mónos, “alone, solitary”) and οἶκος (oîkos, “house”)) and Dioecia comprise the taxa where female and male flowers are borne on the same individual and distinct individuals, respectively.

Linnaeus also introduced the Polygamia category, consisting of the taxa showing hermaphroditic, female and male flowers, not necessarily on a single individual. In a more colorful way, Rousseau [19] specified that in monoecious (Box 2) taxa, although separate sexes are found in the same house (the plant individual), they do not share the same bedroom (the flower).

Based on model-driven character-state reconstructions using an exhaustive sampling of angiosperm flower morphological diversity, Sauquet et al. [20] reconstructed the flower of the most recent common ancestor of angiosperms as bisexual, although (i) the particular combination of states in the reconstructed ancestral flower was found neither in extant nor in extinct taxa so far, and (ii) some authors hypothesized that the ancestral flower might have been unisexual (e.g., [21]). Hypotheses about the evolutionary origin of the bisexual reproductive structure of angiosperms from a monoecious preangiospermous hypothetical ancestor, namely the out-of-male/out-of-female theories, the mostly male model, and the developmental genetic model, were reviewed by Specht and Bartlett [22]. In addition, Sauquet et al. [20] inferred that functional unisexuality (one sex only is functional, the flower possibly being morphologically bisexual) evolved many times independently through the course of angiosperm evolution (see also [23]). This model-based approach also showed that the functionally unisexual flowers of *Amborella* Baill., the sister group of the remaining angiosperms, are derived. Unisexual flowers are largely absent from highly elaborated, animal-pollinated flowers (e.g., Fabales, Orchidales). Exceptions in orchids are *Catasetum* Rich. ex Kunth and *Cycnoches* Lindl. (see Section 8.10.1 in [24]).

The dimorphism (Box 2) of female and male flowers can be total, when unisexuality is a feature of the flower at its inception; the flower is said to be structurally or morphologically unisexual. Alternatively, dimorphism is partial when rudiments of the reciprocal sex are still present; the flower is said to be functionally unisexual.

The diversity of sexual systems (Box 2) in angiosperms (Table 1) is remarkable, depending on the distribution of sexes in different flowers and on different individuals [3,25,26,27]. Diversity in sexual systems can be found at every taxonomic and organizational level, for instance at the genus level (e.g., *Trithuria* (Hydatellaceae) [28]), or even on a single individual (e.g., in *Philodendron solimoesense* A.C. Sm. (Araceae) [29]). Among the 12 species of *Trithuria*, 4 have bisexual flowers, 4 are dioecious (Box 2), and 4 are monoecious. In *Philodendron solimoesense*, distinct genders are longitudinally distributed along the inflorescence. From the base upwards, one observes aborted female flowers, female flowers, developmentally bisexual flowers (carpels and staminodes (Box 2), but sometimes, female organs remain vestigial, or are even aborted), sterile male flowers (with staminodes), male flowers, and aborted male flowers.

**Table 1:** Distribution of functional sexes in the different types of sexual systems.

Flower Gender/Functional Sex			Sexual System When Functional Sex Is Found on:	
Unisexual		Bisexual ^3^		
Female ^1^	Male ^2^		A Single Individual	Distinct Individuals
		✓	Hermaphroditism	
✓	✓		Monoecy ^4^	Dioecy
✓		✓	Gynomonoecy	Gynodioecy
	✓	✓	Andromonoecy	Androdioecy
✓	✓	✓	Trimonoecy ^5^	Trioecy

^1^ Female = pistillate. ^2^ Male = staminate. ^3^ Bisexual = hermaphrodite = perfect = cosexual sensu Barrett [3,11] and Cardoso et al. [12]. ^4^ Monoecy = cosexuality sensu Taylor and Williams [30]. ^5^ Trimonoecy = polygamomonoecy sensu Cardoso et al. [12]. Based on [3,8,16,31,32].

Botanists can rely on several lists and databases of dioecious angiosperm genera and families [10,33,34], and of functionally unisexual species [20]. Lability in this trait is also not seldom, as the sexual system of many plant taxa varies with the environmental conditions preceding the flowering season [35,36,37,38]. The ecological and genetic significance of flower functional unisexuality (e.g., avoidance of inbreeding depression (Box 2)) was addressed in several pivotal studies ([3] and references therein, [39,40]). Terminology relative to flower sexual systems is complex [41], and we provided definitions of a selection of terms in Box 2. A thorough discussion of the nomenclature for angiosperm reproductive systems was presented by Cardoso et al. [12]. The current terms in the field of sexual systems belong to different levels: structural, developmental, and physiological. Hence, a uniform system that would be applicable to all levels could hardly be formulated [24].

The development of unisexual flowers has been—more or less precisely—described in a large number of taxa, sampling the diversity of floral phenotypes observed in extant angiosperms, in studies focusing on floral ontogeny, on the evo-devo of unisexuality, or on the genetic and chromosomal bases of unisexuality (for review, see [42,43]). We review here such developmental studies, aiming at characterizing the diversity of ontogenic pathways leading to functionally unisexual flowers. In addition, we quantitatively compare the developmental rate of the sexual organs in functionally unisexual flowers, in a non-exhaustive sampling of angiosperms with contrasted floral morphologies. Eventually, we provide recommendations to help plant evo-devo researchers and botanists addressing macroevolutionary and ecological issues to more precisely select the taxa, the biological material, or the developmental stages to be investigated.

## 2. Meristem Development in Taxa with Unisexual Flowers, in an Evolutionary Framework

### 2.1. Morphological and Anatomical Description of Functionally Unisexual Flowers

**Figure 1:**
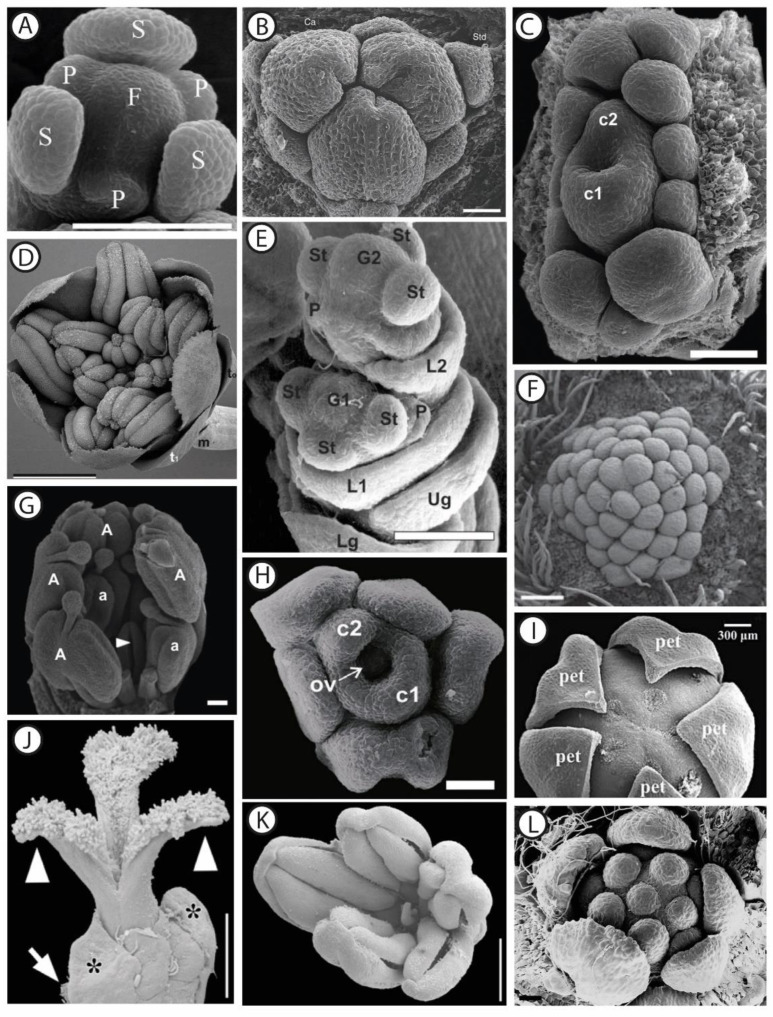
SEM micrographs of dissected male and female flower buds at different developmental stages, in selected angiosperm taxa. (**A**) Female flower of *Stephania japonica* Thunb. (Miers) (Menispermaceae) showing sepals (S), petal primordia (P), and the floral apex (F). Scale bar: 150 µm. Adapted with permission from Ref. [46]. (**B**) Female flower of *Gaussia attenuata* (O.F. Cook) Becc. (Arecaceae) showing the tricarpellate (Ca) gynoecium and the sterile androecium consisting of six staminodes (Std). Scale bar: 50 µm. Adapted with permission from Ref. [47]. (**C**) Female flower of *Ampelocera glabra* Kuhlm. (Ulmaceae) showing two united carpels (c1 and c2) surrounded by 11 staminodes at different developmental stages. Scale bar: 100 µm. Adapted with permission from Ref. [48]. (**D**) Male flower of *Amborella trichopoda* Baill. (Amborellaceae) showing tepals (t_1_) surrounding stamens at an early anthetic stage. t_0_: transverse bracts, m: median bract on peduncle. Scale bar: 1 mm. Adapted with permission from Ref. [49]. (**E**) Spikelet of *Panicum maximum* Jacq. (Poaceae) showing the initiation of the gynoecia (G1 and G2) and the beginning of the elongation of glumes. Both florets are initially bisexual, and while the distal (lower) floret remains hermaphroditic up to anthesis, the proximal one develops as a male floret by abortion of the gynoecium primordium. Lg: lower glume, Ug: upper glume; L1: lemma of the proximal floret; St: stamen primordium, G1: gynoecium primordium of the proximal floret, P: palea, G2: gynoecium primordium of the distal floret. Scale bar: 100 µm. Adapted with permission from Ref. [50]. (**F**) Male flower of *Pseuduvaria indochinensis* Merr. (Annonaceae) showing the central part of the floral meristem covered with numerous stamen primordia. Scale bar: 200 µm. Adapted with permission from Ref. [51]. (**G**) Male flower of *Stryphnodendron adstringens* (Mart.) Coville (Fabaceae) showing a carpellodium (arrowhead pointing to cleft) surrounded by stamens (a and A). Scale bar: 500 µm. Adapted with permission from Ref. [52]. (**H**) Female flower of *Celtis iguanaea* (Jacq.) Sarg. (Cannabaceae). The large (c1) and small (c2) carpels are surrounded by staminodes. Ov: ovule. Scale bar: 50 µm. Adapted with permission from Ref. [53]. (**I**) Female flower of *Schefflera venulosa* (Wight & Arn.) Harms (Araliaceae) showing the pentamerous gynoecium surrounded by petals (pet). Scale bar: 300 µm. Adapted with permission from Ref. [54]. (**J**) Anthetic female flower of *Pistacia lentiscus* L. (Anacardiaceae). The ovary is unilocular, as only one of the three carpels is fully developed at anthesis. Asterisks indicate two sepals (bracts? (sic)). White arrow points to floral subtending bract. Arrowheads point to the pair of sterile stigmas. Scale bar: 0.5 mm. (**K**) Male flower of *P. lentiscus* showing five stamens and a central aborted gynoecium. Scale bar: 0.8 mm. Both (**J**) and (**K**) adapted with permission from Ref. [55]. (**L**) Male flower of *Croton schiedeanus* Schltdl. (Euphorbiaceae), showing the pentamerous calyx surrounding six stamens (note that the central organ is a developing stamen). Scale bar: 20 µm. Adapted with permission from Ref. [56].

**Figure 2:**
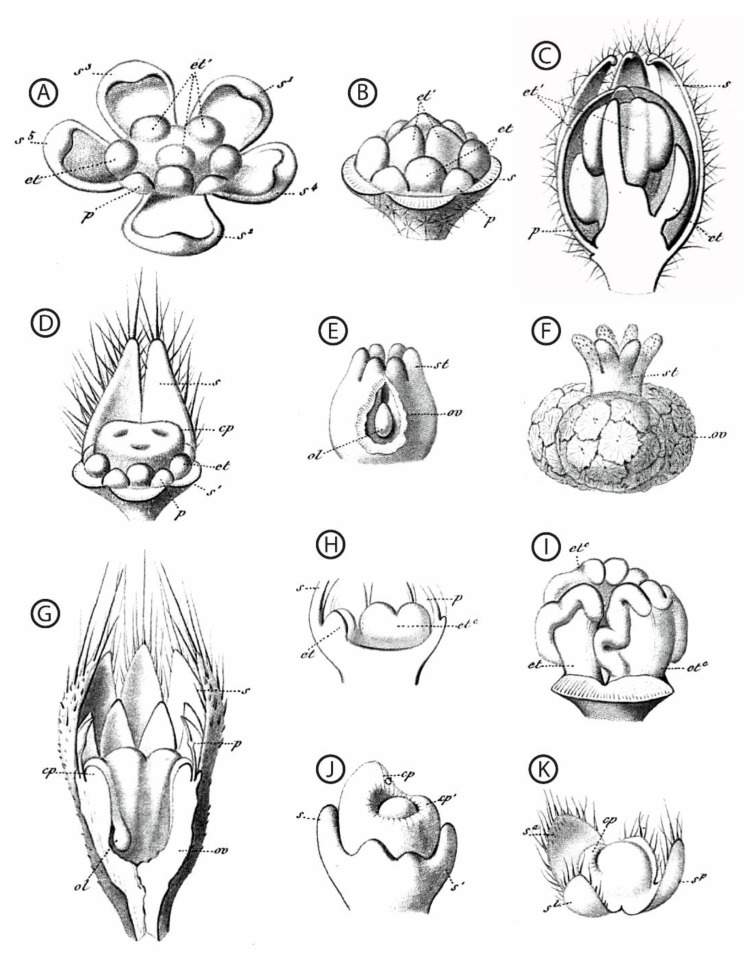
Hand-drawn dissected floral buds and floral organs, from a sample of taxa with functionally unisexual flowers. All drawings are from [57]. (**A**–**F**): *Chrozophora tinctoria* (L.) (**A**). Juss. (Euphorbiaceae); (**A**–**C**): successive developmental stages of a male flower, highlighting the central position of the androecium; (**D**): dissected bud of a female flower, showing the young gynoecium surrounded by stamen primordia; (**E**,**F**): successive developmental stages of the gynoecium. (**G**): *Sicyos angulatus* L. (Cucurbitaceae), young female flower. (**H**,**I**): *Cucurbita pepo* L. (Cucurbitaceae), two successive developmental stages highlighting the position of the androecium at the center of the receptacle. (**J**): *Ficus carica* L. (Moraceae), female flower. (**K**): *Urtica cannabina* L. (Urticaceae), female flower. First and second taxa spelled as *Chrozophora tinctoria* and *Sicyos angulata*, respectively, in [55]. s: sepal, p: petal, et: stamen, cp: carpel, st: style, ov: ovary, ol: ovule. Superscripts give details about the shape, position, or initiation sequence of the organs; they are not essential here.

Describing the morphology of functionally unisexual flowers basically consists in analyzing their ontogeny, aiming at identifying the stage at which the development of the non-functional organs is arrested. Detailed observations have contributed to our knowledge, based on scanning electron microscopy studies (Figure 1) or simply using a binocular (Figure 2). In a second step, investigating floral anatomy allows testing the hypotheses whether the abortive organs are vascularized, or whether meiosis is completed (e.g., [44,45]).

Describing the sexual system within a lineage requires conducting a comparative study within a taxon, looking at a representative sample of individuals and populations throughout its distribution area, as it is known that sexual systems can be influenced by seasonal and ecological constraints (e.g., [35,58]).

It has to be noted that some dioecious taxa are only known from flowers from a single sex, in cases where individuals bearing flowers from the opposite sex have been found neither in the wild nor in herbaria and botanical gardens (e.g., [59], see also Box 3).

Box 3 **Sampling of angiosperm taxa from the fossil record exhibiting unisexual flowers, presented in chronological order. The numbers found in the linear sequence of fossils are reported on a geologic time scale (adapted with permission from Ref. [60]) and on a phylogenetic tree of angiosperms (adapted with permission from Ref. [61]). Note that this list does not include any instance of a flower of a given sex showing any sterile or rudimentary organ of the reciprocal sex.**

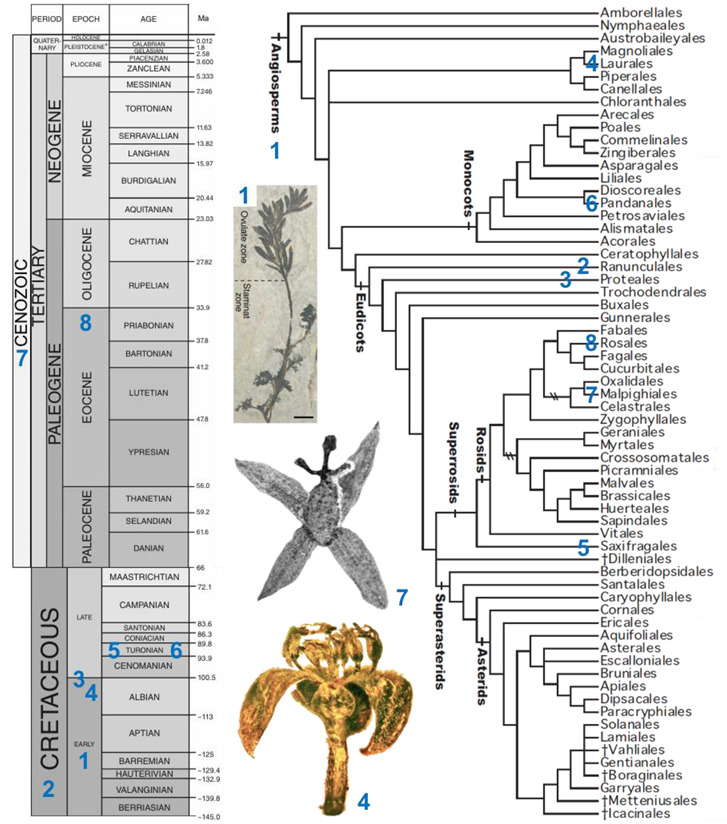

1. *Archaefructus sinensis* Sun, Dilcher, Ji et Nixon (Archaefructaceae, Early Cretaceous, China, see associated picture). Flowers are interpreted as unisexual ([62], but see [63]); female and male flowers show two carpels and two stamens, respectively.
2. *Teixeira lusitanica* von Balthazar, Pedersen and Friis (affinities with Ranunculales, Cretaceous, Portugal). A single male flower including 20 stamens with basifixed anthers was described [64].
3. Platanaceae sp. (Proteales, Middle Cretaceous to Albian, USA). Pentamerous male (with five stamens) and female (with five carpels) flowers were described [65].
4. *Cascolaurus burmitis* Poinar (Lauraceae, Laurales, Upper Albian, Myanmar, see associated picture). The male flower presents three whorls of three stamens each with, probably, nectar glands located on the stamens of the innermost whorl [66].
5. *Microaltingia apocarpela* Zhou, Crepet, and Nixon (affinities with Hamamelidaceae, Saxifragales, Turonian, USA). Female flowers show a gynoecium consisting of a semi-inferior bilocular ovary, a style, and a capitate stigma [67].
6. *Mabelia connatifida* Gandolfo, Nixon, et Crepet (Triuridaceae, Pandanales, Turonian, USA). Male flowers include a trimerous whorl of antetepalous stamens [68].
7. *Pseudosalix handleyi* Boucher, Manchester, and Judd (Salicaceae, Malpighiales, Cenozoic, USA, see associated picture). The gynoecium of the female flower consists of a pistil with a single ovary. The androecium of male flowers includes ca. 30 stamens [69].
8. *Prunus s.l.* (Rosaceae, Rosales, terminal Eocene, Ukraine). A fossil male flower, with 24 free stamens, was discovered [70].

### 2.2. Diversity of Ontogenic Pathways Leading to Functionally Unisexual Flowers

Two main ontogenic pathways leading to functionally unisexual flowers have been proposed, namely the “unisexual by abortion” pathway (type I, Box 2) and the “unisexual from inception” pathway (type II, Box 2; [17,26,71,72]. Authors propose that a hermaphroditic floral morphology could evolve towards a structurally unisexual flower through homeosis, and that type-I flowers could evolutionarily lead to type-II flowers through heterochrony.

**Figure 3:**
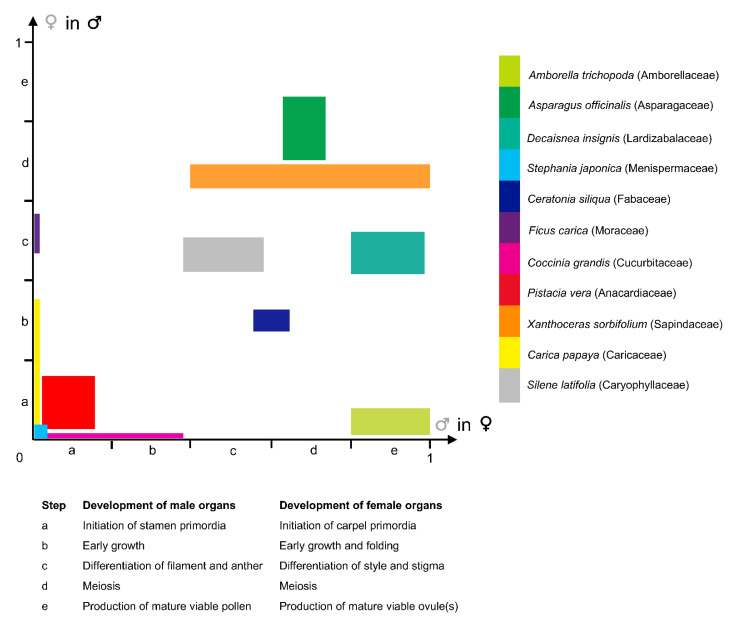
Graphical synthesis in a two-dimensional morphospace of the diversity of developmental pathways observed in species with functionally unisexual flowers. Developmental rate of organs of a given sex, in a flower bud of the opposite—functional—sex, estimated from published data, from 0 (absence of organ) to 1 (fully-developed and functional organ), and broken up in five steps (see Table within the Figure). X-axis: Developmental rate of male organs in functionally female flowers. Y-axis: Developmental rate of female organs in functionally male flowers. Developmental data for selected taxa were taken from the following publications: *Amborella trichopoda* Baill. (Amborellaceae) [44]; *Asparagus officinalis* L. (Asparagaceae) [76]; *Decaisnea insignis* (Griff.) Hook. f. & Thomson (Lardizabalaceae) [77]; *Stephania japonica* (Thunb.) Miers (Menispermaceae) [46]; *Ceratonia siliqua* L. (Fabaceae) [72]; *Ficus carica* L. (Moraceae) [78]; *Coccinia grandis* (L.) Voigt (Cucurbitaceae) [79]; *Pistacia vera* L. (Anacardiaceae) [80]; *Xanthoceras sorbifolium* Bunge (Sapindaceae) [45]; *Carica papaya* L. (Caricaceae) [81]; *Silene latifolia* Poir. (Caryophyllaceae) [75].

Considering that the arrest in development can occur at any step of the developmental sequence of the stamens and carpels (between the initiation of primordia until meiosis completion and production of viable functional gametes; Figure 3; [71]), and by diverse mechanisms [17,73], there are multiple developmental trajectories leading to floral unisexuality associated with the apparent functional convergence (e.g., [74]). Figure 3 illustrates the fact that the development of the organs of the non-functional sex occupies most of the 2D morphospace circumscribed by both axes of the graph. For instance, in the dioecious *Silene latifolia* Poir. (Caryophyllaceae), the development of the female and male organs is similar to those of a bisexual flower during the first developmental stages. At the sixth developmental stage (on a total of 12), five carpel primordia are initiated in the female flower while in the male flower a single carpel primordium is visible. Stamen morphogenesis continues in both male and female flowers until stage 7, which is the stage when the non-functional organ stops its development [75]. In contrast with *S. latifolia*, in which female and male floral ontogenies diverge starting from the same developmental stage, a gender-specific type of development is observed in other species such as *Celtis iguanaea* (Jacq.) Sarg. (Cannabaceae). In this species, floral functional unisexuality occurs earlier in the male flower (gynoecium development stops before carpel elongation and a pistillode (Box 2) is formed) than in the female flower (androecium development stops before pollen maturation in the staminodes) [53].

A large morphological diversity of functionally unisexual flowers exists, differing by the developmental rate of the non-functional sex, and has been described in the botanical literature. Flowers can be structurally unisexual, when organs of the opposite (non-functional) sex are not initiated (e.g., *Stephania* Lour. (Menispermaceae); Figure 3; [46]; and many Arecaceae genera showing male flowers with a completely aborted gynoecium [13]; see also [72,82] for lists of taxa belonging to this category). Floral development of some taxa showing type-II flowers is shown in Payer [57] for *Cannabis* L. (Cannabaceae), *Ficus* L. (Moraceae), *Typha* L. (Typhaceae), and *Urtica* L. (Urticaceae) (Figure 2). Additional examples of unisexual-from-inception flower development are presented by Sattler [83] for *Quercus* L. (Fagaceae), *Juglans* L. (Juglandaceae), and *Populus* L. (Salicaceae), for instance. In type-II male flowers, the center of the meristem may be (i) occupied by stamens (e.g., the synandrium of male *Stephania* flowers is located at the very center of the meristem [46]), (ii) filled with rudimentary female organs, or (iii) an empty space. Alternatively, flowers can be functionally unisexual, bearing more or less developed organs (hence called rudimentary or vestigial) of the opposite sex (e.g., *Pennantia* J.R. Forst. & G. Forst. (Pennantiaceae) [84]). Based on a study of the literature, it seems that unisexual flower morphologies are very diverse throughout the angiosperms, and that similar morphologies (with comparable developmental state of the organs of the non-functional sex) can be found in phylogenetically distant lineages (Figure 3). We highlight in a selection of paleobotanical studies having described unisexual fossil flowers that, although the diversity in floral organization is remarkable, there were no instances of flowers of a given sex including any sterile or rudimentary organ of the reciprocal sex (Box 3).

Identifying the developmental stage at which the differentiation between functionally female and male flowers occurs in a given species requires comparing the ontogenic sequences of flowers from both sexes and determining the developmental landmark after which development diverges in each sex.

The development of both staminate and pistillate (Box 2) flowers in the (mostly) dioecious genus *Carica* L. is identical up to stamen initiation ([81], Figure 3). While in staminate flowers, gynoecium grows into a central pistillode, probably functioning as a nectary, pistillate flowers have no traces of stamens.

In some cases, precise morphological landmarks are identified, pinpointing the stage at which the development of each sex diverges. For instance, in *Ceratonia siliqua* L. (Fabaceae), when stamen height reaches c. 350 μm, the carpel stops growing in the male flower. It remains as a slight point at the center and ovules are absent. Functionally female flowers have totally abortive stamens [72]. In other cases, the developmental sequence is broken up in several stages and the time of morphological differentiation between both sexes is hence placed in a precise framework of developmental events. Zhou et al. [45] showed that early developmental stages of the morphologically andromonoecious (Box 2) *Xanthoceras sorbifolium* Bunge (Sapindaceae) were identical in both sexes. Male and morphologically bisexual flowers started to differentiate at stage 8 (9 stages in all) when the style developed further in bisexual flowers, but not in male flowers (Figure 3). In staminate flowers, ovule development was arrested after the formation of megaspore mother cells or during meiosis. Anther development was aberrant in bisexual flowers: they did not dehisce, the filament failed to elongate in most flowers, and the pollen was not functional.

### 2.3. Expression of Gender

In monoecious species, female and male genders can be expressed simultaneously (synchronously) or consecutively in different flowers on the same individual. For instance, in the monoecious *Acer campestre* L. (Sapindaceae), some individuals are protogynous (functionally female (their anthers do not dehisce) flowers open first, followed by the functionally male (gynoecium is rudimentary) flowers), others are protandrous [16]. Temporal separation of gender expression could also be observed in a single flower in dichogamous (Box 2) species (e.g., *Lepianthes peltata* (L.) Raf. ex R.A. Howard (Piperaceae), [24] (p. 193). The likelihood of self-pollination is reduced in such consecutive monoecious or dichogamous species.

In some angiosperm taxa, the functional sex can be expressed and displayed in such a way that it carries a secondary function (the primary function being reproduction), namely mimicry. In the chestnut (*Castanea* Mill. (Fagaceae)), female flowers have erect styles resembling stamens from male flowers; this is a probable case of intersexual mimicry [85]. Thien et al. [86] suggested that *Amborella* is another case of automimicry (Box 2), as functionally female flowers mimic male flowers to attract pollen-feeding insects. Female *Amborella* flowers are structurally bisexual, with 1–2 staminodes in which sporogenous tissue is differentiated but where meiosis does not take place [44].

### 2.4. Phenotypic Plasticity of Sexual Systems and Sexual Instability

In many taxa, the organs of the non-functional sex show a wide developmental lability. Plasticity in gender expression means that sexual function changes adaptively during each individual’s lifetime [3,11]. Depending on the individual, the season, or for no obvious reason, the organs of the usually non-functional sex develop until they reach meiosis and become functional [3,10]. Exceptions to strict dioecy are hence not seldom.

An extreme form of sexual plasticity is environmental sex determination [87,88,89], which occurs in *Acer* L. [90] and Catasetinae (Orchidaceae, [91]), among other taxa [10]. In *Xanthoceras sorbifolium* for instance, proportion of functionally male and female flowers varied from year to year, from tree to tree, and from inflorescence to inflorescence [92].

Within a single species, sexual instability, or variation in sexual system, can be recorded. Sexual instability is a common phenomenon in dioecious plant species, and many exogenous, environmental, and demographic factors are known to affect sex ratios, including plant hormones [93], temperature [94], pathogens [95], timing of seed set [96], and population structure [97]. Some of these factors strongly suggest the involvement of epigenetic mechanisms operating in sex determination. A particularly striking example of intraspecific variation in sexual systems occurs in the ruderal weed *Mercurialis annua* L. (Euphorbiaceae). Dioecious, monoecious, and androdioecious (Box 2) populations occur in different parts of Europe, and recent studies indicate that contrasting ecological and demographic conditions play a critical role in determining which sexual system is maintained [98]. In *Amborella trichopoda* Baill, two types of sexual instability were described [99]. First, authors noted a small proportion (approximately 1%) of complete sex change events in a population of young plants grown from seed (see also [49]). Second, they found a variable proportion of bisexual flowers on some predominantly male individuals in both ex situ and in situ populations.

## 3. Molecular Bases of Floral Unisexuality

As several reviews dealing with the molecular bases of floral unisexuality have been published [39,100,101,102,103,104,105,106], we present here a few illustrative cases only.

The first studies investigating the molecular bases of heritability and establishment of sex in plants were carried out at the beginning of the 20th century, at a time when biologists became familiar with Mendelian genetics [107]. Based on the analysis of sex ratios of the offspring from reciprocal pollinations between dioecious *Bryonia dioica* Jacq. (Cucurbitaceae) and monoecious *B. alba* L., the presence of sex chromosomes (or allosomes: chromosomes carrying sex-determining genes) with a Mendelian-type segregation was proposed [108,109,110]. In *Silene dioica* (L.) Clairv. and *Vitis* L., genes responsible for the expression of sexually-related morphological features (e.g., leaf form) were identified on such so-called sex chromosomes ([111,112,113,114]; reviewed in [115]). Later in the 20th century, in an attempt to explain how species can give rise to individuals of different sexes and based on the assumption that dioecious species originated from hermaphroditic lineages, Charlesworth and Charlesworth [116] proposed a model based on two fundamental principles: (1) the existence of at least two genes (one that inhibits the development of the ovules and another that prevents the development of pollen) and that (2) these two genes are located in the same allosomal region.

X and Y sex chromosomes have been identified in many plant species ([39,117], but [118], for the description of the ZW chromosome system in *A. trichopoda*). In addition, the establishment of the sexual phenotype seems taxon-specific to a large extent. In *Rumex acetosa* L. (Polygonaceae) and in *Humulus lupulus* L. (Cannabaceae), gender is established at very early stages of development and depends on the ratio between the number of X chromosomes and autosomes: a ratio of 0.5 or less results in male plants; a ratio of more than 1.0 results in female plants. In these type-II species, the early establishment of gender during floral ontogeny is controlled by the X chromosomal information, and the unisexual flowers show no rudimentary organs of the reciprocal sex [119,120]. In *Silene latifolia*, the Y chromosome includes two regions that are highly important in sex determination: one region inhibiting the development of gynoecium and another that promotes the development of androecium. In this case, XX individuals are females while individuals with one Y and up to three X chromosomes are males. Although DNA methylation on the X chromosomes was shown to play an important role in sex determination, the process by which the X chromosome is involved in the development of female sex organs in *S. latifolia* flowers has not been clearly established [121,122].

In addition to sex determination through allosome-based promotion or inhibition of the sexual organs, the control of floral organ identity during development is fundamental in sexual determination. The genetic control of the floral organ identity has been explained by a model composed of three main functions: the A-function which, acting alone, determines sepal identity; the B-function which, acting together with A-, determines petal identity; and the C-function which, acting together with B-, determines stamen identity and alone determines carpel identity [123]. Following this model, it is expected that B- and C-function gene expression is affected during the floral development of species exhibiting unisexual flowers. Indeed, in most of the species with sex chromosomes, sexual determinism appears to be directly controlled through the activity of the genes belonging to both B- and C-functions. *Rumex acetosa* staminate and pistillate flowers are unisexual and do not include any developed organs from the non-functional sex. In this species, *PLENA*-like (*RAP1*) (C-function gene homologous of *Arabidopsis* Heynh. *AGAMOUS*), responsible for the identity of male and female organs, shows a differential expression in flowers of each gender: after an initial expression in both stamen and carpel primordia, the expression is reduced in the primordia that will not develop, i.e., the primordia of the organs of the non-functional sex [119]. The meristematic regions where *Silene latifolia MADS 2* (*SLM2*) and *SLM3* (B- function genes homologous of *PISTILLATA* (*PI*) and *AP3* respectively) are expressed differ in size between male and female flowers. In addition, it was shown that the Y chromosome, promoting androecium development in this species, controls the activity of B- and C-function genes [122]. In *Spinacia oleracea* L., the *SpAG* (C-function) is involved in anther maturation and in the earlier termination of floral meristem in male flowers. Suppression of the activity of the B- function genes (*SpAP3* and *SpPI*) allows a feminization of the spinach flower [124]. Interestingly, Shephard et al. [120] reported in *H. lupulus* the presence of organs with intermediate sexual characteristics that may indicate a disturbance in the limits of expression of B- and C-function genes.

Although floral organ identity genes play a fundamental role in floral sexual determination, they are not sufficient to fully explain the diversity of mechanisms underlying unisexuality in flowers. In contrast to type-II species, the genetic modifications linked to organ degradation in *Cucumis sativus* L. seem to be due to intra-whorl modification rather than identity modification. Only the well-delimited portions of the stamens and ovaries responsible for producing mature gametes are aborted, and not the whole androecium or gynoecium [125].

In addition to chromosomal composition and floral organ identity genes expression, different studies have established the influence of hormones on floral sexual determination. Evidencing the role of hormones in this respect could be done through hormone inoculation or through genetic control of hormone biosynthesis. Hormone biosynthesis is directly related to factors as day length or temperature modifications indicating that flower sexual determination can be controlled by extrinsic factors. In *Zea mays* L., gibberellins and the *DWARF* and *ANTHER*-*EAR* genes, involved in gibberellin biosynthesis, play a key role in flower feminization [100]. In *H. lupulus*, the same hormone seems to control the development of secondary characters related to gender, such as inflorescence branching [120,126]. Cytokinins and auxins are correlated in sex determination of male and female flowers, respectively, in *Mercurialis* L. [100]. In contrast, in *H. lupulus*, the latter seems to participate in the masculinization of the flower, allowing stamen development [127], in opposition to *Populus tomentosa* Carrière, where auxins and ABA affect early male flower development through the activity of *GA20ox*, *SAUR39*, and *CKX3* genes [128]. In *Morus rubra* L. and *M*. *alba* L., phytoestrogens likely regulate the expression of genes influencing the development of female reproductive structures [129]. In *Cucumis melo* L. and *C*. *sativus*, ethylene production, mediated by *ASC1*, plays a role as an endocrine messenger in the phloem. The sexual determination activity of this hormone depends on the developmental stage at which it is produced in the plant individual [130,131,132].

## 4. Conclusions

Depending on the field of study, researchers might focus on flower morphology without testing whether both sexes within a flower are functional, or might deal with monoecious or dioecious taxa without documenting precisely the potential diversity of sexual systems in the field (or based on herbarium collections). Alternatively, botanists addressing macroevolutionary and ecological issues might only focus on the functional sex (e.g., [133]). Based on this statement, we advocate (i) aiming at a thorough characterization of the sexual system of the taxon under scrutiny, interpreting the development of both structural and functional sexes, and (ii) including the sister lineage in any evolutionary/developmental study of a taxon presenting unisexual flowers.

The precise description of the disparity of flower sexual organs in a given taxon and the identification of the developmental stage reached by the non-functional sexual organs are key to accurately select the taxa, the biological material (populations, individuals, single flowers), or the developmental stages to be investigated.
